# Computer vision syndrome among students during remote learning periods: harnessing digital solutions for clear vision

**DOI:** 10.3389/fpubh.2023.1273886

**Published:** 2023-11-09

**Authors:** Muna Abed Alah, Sami Abdeen, Nagah Selim, Layla AlDahnaim, Iheb Bougmiza

**Affiliations:** ^1^Community Medicine Department, Hamad Medical Corporation (HMC), Doha, Qatar; ^2^Community Medicine Department, Primary Health Care Corporation (PHCC), Doha, Qatar; ^3^Public health and preventive medicine Department, Cairo University, Giza, Egypt; ^4^School Health Services and Programs, Primary Health Care Corporation (PHCC), Doha, Qatar; ^5^Community Medicine Department, College of Medicine, Sousse University, Sousse, Tunisia

**Keywords:** computer vision syndrome, digital eye strain, remote learning, COVID-19, school closures, students

## Abstract

**Aim:**

This study aimed to assess the prevalence of Computer Vision Syndrome (CVS) among children and adolescents in Qatar during the period of remote learning and explore the associated factors and discuss some digital health remedies that might reduce the risk.

**Methods:**

We conducted an analytical cross-sectional study between June and August 2022 by collecting data via telephone interviews with parents of selected students utilizing the Computer Vision Syndrome Questionnaire (CVS-Q).

**Results:**

We completed 1,546 interviews. The mean age of the students was (11 ± 2), male: female ratio was almost 1:1. About one quarter (368, 23.8%) of parents reported a previous diagnosis of visual disturbances among their children with over 88% of them wearing eyeglasses or medical contact lenses. The prevalence of CVS in our sample was about 8% (95%CI: 6.8–9.6). Mother’s employment, having positive history of visual disturbances, and excess screen time were found to be significant predictors of CVS.

**Conclusion:**

Health care providers in collaboration with teachers should provide parents with evidence-based strategies to prevent or minimize the digital eye strain among students. In the landscape of remote learning, the implementation of digital remedies emerges as a proactive approach to mitigate the risk of digital eye strain.

## 1. Introduction

COVID-19 has been the focus of governmental decisions worldwide since the World Health Organization declared it a global pandemic in March 2020. Many countries were forced to impose swift restrictive measures to limit the spread of the virus including the closure of shopping malls, working from home, and closure of schools with shifting to remote learning (1, 2). The direct consequences of COVID-19 were less severe among children and adolescents in terms of mortality and morbidity (3, 4). However, the indirect effects on their lifestyle and health were tremendous. The literature showed that the containment measures associated with the pandemic resulted in a reduction in physical activity, increased sedentary behaviors, and screen time among children and adolescents (5–9). Due to the mandated restrictions during school closures, individuals of various ages have reported heightened levels of anxiety, anger, and stress (10, 11). Contemporary studies indicate that during infectious outbreaks, those in isolation or quarantine encounter multiple stressors including concerns about personal health, apprehensions about transmitting the disease to others, feelings of boredom, frustration, and a profound sense of isolation (12–14). This sense of detachment arises from confinement, disruption of regular routines, and diminished social and physical interactions with others (12, 13, 15, 16). Such factors can contribute to an increased reliance on digital devices, leading to elevated screen time. In fact, during the pandemic-induced school closures, social media and technology served as adolescents’ primary form of socialization (17). But the way adolescents engaged with these platforms greatly influenced their emotional well-being, with active engagement often leading to feelings of happiness for some and stress for others (17). Additionally, children’s screen time activity was greatly influenced by the heightened parents stress levels during the pandemic that resulted in behavioral difficulties and increased digital device use (18). The increased use of digital devices during the home confinement measures for remote learning, entertainment, or social communications resulted in deterioration of visual health with either induced or exacerbated visual disturbances such as visual fatigue, myopia, and dry eye among others (19). The increased screen time brought about by the increased dependence on digital devices resulted in computer vision syndrome (CVS), also referred to as digital eye strain (DES) (20) which describes a group of eye and vision-related problems that result from prolonged computer, tablet, e-reader, and smartphone use (21). In some studies, the prevalence of CVS among children and adolescents exceeded 50% (20, 22, 23). In Saudi Arabia, about 70% of children aged 3 to 18 years reported digital eye strain symptoms during lockdown measures (24). Recent research underscores the role of sociodemographic factors, especially age and gender, in influencing the prevalence of CVS, particularly during the COVID-19 era (25). Studies have shown higher prevalence among younger ages and females (25, 26). These findings stress the significance of investigating such factors in understanding the development of CVS.

Symptoms of CVS have the potential to impede essential academic activities such as reading, professional tasks, and computer utilization (27). Vision, being integral to a child’s holistic health and well-being, significantly influences their physical, cognitive, social, and emotional growth. Notably, enhanced visual capabilities have been directly correlated with academic excellence, emphasizing the criticality of optimal visual performance within educational environments (28). The transition to remote learning and the shift to home-based study can intensify the effects of CVS. This is because home settings may lack the ergonomic considerations found in classrooms or computer labs, which can result in suboptimal posture and screen placement, thereby heightening the risk of developing CVS.

Students in Qatar shifted to remote learning in March 2020. This was followed by a period of blended learning combining online and face-to-face classes on alternative basis. However, it was only until September 2021 that schools were reopened again with a full resumption of face-to-face learning. In this study, we aimed to assess the prevalence of CVS among students in Qatar during the remote learning period and explore the associated factors and discuss some digital health remedies that might reduce the risk. To the best of our knowledge, studies assessing the CVS among this segment of the population are limited and this is the first in Qatar to address this issue.

## 2. Methods

We conducted an analytical cross-sectional study targeting students in Qatar between 8–15 years of age. The data collection took place between June and August 2022.

### 2.1. Study procedure and the sampling technique

The sampling frame for this study was extracted from the national electronic health record system in Qatar. Using a stratified random sampling method, we selected a proportionate number of students stratified by age group and sex. The data were collected from the parents of selected participants by telephone interviews.

### 2.2. The data collection tool and outcome measures

The presence of CVS was assessed using the Computer Vision Syndrome Questionnaire (CVS-Q) which is a valid and reliable tool to assess the presence and severity of symptoms of digital eye strain developed by Segui et al. (29). Parents were asked to indicate whether their children complained or suffered from any digital eye strain symptoms during the period of COVID-19-related schools’ closure and remote learning. The frequencies and intensities of 16 digital eye strain symptoms were collected on a five-point Likert scale (Never, occasionally of moderate intensity, occasionally of severe intensity, always of moderate intensity, always of severe intensity). The score for each symptom was calculated by multiplying the code of the frequency by the code for the intensity of the symptom [where frequency: never = 0, occasionally = 1, often or always = 2, and intensity: moderate = 1, sever intensity = 2], and the result of frequency × intensity was recoded as 0 = 0; 1 or 2 = 1; 4 = 2. Then, the total score for each student was calculated by summing the individual scores by applying the following formula:

$\begin{array}{c} \text{Total}\;DES\;\text{score}=\Sigma \;\left(\text{frequency}\;\text{of}\;\text{symptom}\;\text{occurrence}\right)\\ \;\times \left(\text{intensity}\;\text{of}\;\text{symptom}\right) \end{array}$

According to Segui et al. (29), students with total scores of six or more points were considered suffering from CVS. The scores were further categorized as mild (score = 6–12), moderate (score = 13–18), and severe (score = 19–32).

In addition to the CVS-Q, we assessed the sociodemographic characteristics of the students (nationality, parental ages, educational levels, mother’s employment status…), and other relevant information such as the status of wearing eyeglasses or medical contact lenses, and history of visual disturbances like refractive errors or previously diagnosed eye disease. Moreover, parents were asked to indicate the average screen time of their children before and during school closures (time spent viewing screens using digital devices like smartphones, computers, and laptops) excluding the time spent in online classes (as the duration of the online classes was almost the same for all students during the closure period) and to indicate the most commonly used digital device for online classes.

### 2.3. Data analysis plan

We analyzed the data using IBM SPSS Statistics for Windows, version 26.0. Armonk, NY: IBM Corp. Categorical data were presented as percentages, and continuous data as mean and standard deviation. The Chi-square or Fisher Exact tests were used to compare categorical outcomes between groups, while the independent Student’s *t*-test or Mann–Whitney U test was used to compare continuous and ordinal outcomes as appropriate. To identify potential CVS predictors, we performed a logistic regression analysis selecting the variables based on existing literature or having value of *p* less than 0.25 in bivariate analysis. The associations between risk factors and outcomes were presented as adjusted odds ratios (AORs) and 95% confidence intervals (95%CIs). The goodness of Fit was assessed using the Hosmer–Lemeshow test. value of *p*s less than 0.05 were considered significant.

### 2.4. Ethical approval

Ethical approval was obtained from the institutional review boards (IRBs) of Hamad Medical Corporation (MRC-03–21-895) and the Primary Health Care Corporation (PHCC/DCR/2021/09/059).

## 3. Results

We completed 1,546 interviews with the parents of selected students. The mean age of the students was (11 ± 2), male to female ratio was almost 1:1. Of the total sample, 572 (37%) were local students, and the rest were expatriates. About one quarter (368, 23.8%) of parents reported a previous diagnosis of visual disturbances among their children, with over 88% of them wearing eyeglasses or medical contact lenses (Table 1). Female students had significantly higher proportions of visual disturbances (*p* = 0.003). Similarly, higher proportions were also found among those 12 years and older compared to the younger ones (*p* < 0.001).

**Table 1:** Sociodemographic characteristics of the included students and background information.

Characteristic	No (%)	
Student’s age category	8–11 years	845 (54.7)
	12–15 years	701 (45.3)
Gender	Female	777 (50.3)
	Male	769 (49.7)
Nationality	Expatriates	974 (63.0)
	Locals (Qatari)	572 (37.0)
Number of siblings	3 or less	740 (47.9)
	4–6	655 (42.4)
	>6	151 (9.8)
History of visual disturbances	No	1,178 (76.2)
	Yes	368 (23.8)
Wearing eyeglasses	No	1,219 (78.8)
	Yes	327 (21.2)
Mother’s age category	<35	297 (19.6)
	35–44	902 (59.5)
	45–54	303 (20.0)
	55 or more	13 (0.9)
Father’s age category	<35	68 (4.5)
	35–44	688 (45.7)
	45–54	568 (37.8)
	55 or more	180 (12.0)
Mother’s education	No formal education	76 (4.9)
	Primary school level	101 (6.5)
	preparatory school level	126 (8.2)
	Secondary/ high school level	466 (30.1)
	College or higher	777 (50.3)
Father’s education	No formal education	40 (2.6)
	Primary school level	77 (5.0)
	preparatory school level	137 (8.9)
	Secondary/ high school level	414 (26.8)
	College or higher	878 (56.8)
Mother’s employment status	Employed	711 (46.0)
	Not employed	835 (54.0)
Type of digital device that was used for online classes most of the time	Computers (desktops or laptops)	394 (25.5)
	Notepad/iPad/Tablet	663 (42.9)
	Smartphone	489 (31.6)

**Figure 1:**
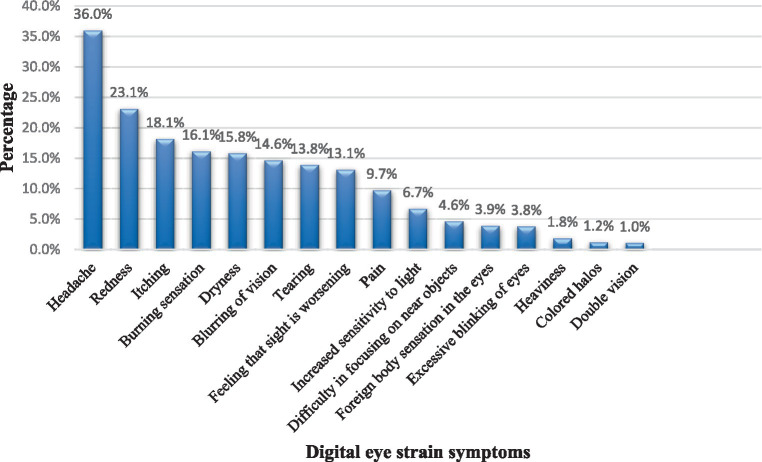
The distribution of digital eye strain symptoms among governmental school students during school closures in Qatar as reported by parents.

Excluding the time spent attending online classes, the mean screen time for the whole week during schools’ closure was found to be 29 ± 14 h/week with an average of 4 ± 2 h/day representing a significant increase in the screen time when compared to before the closure which was about 17 ± 10 h (*p* < 0.001). The most used digital device during online classes was Notepad/iPad/Tablet (42.9%) in the total sample, and both sexes. However, smartphones were the most common type reported among those 12 years or older. The prevalence of CVS/DES in our sample was found to be 8.2% (127 out of 1,546), (95%CI: 6.8–9.6). However, over 50% of students reported at least one visual symptom during school closures. The most reported symptoms were headache 556 (35.9%), eye redness 357 (23.1%), and eye dryness 244 (15.8%) as shown in Figure 1. The scores of the included students ranged from 0–18. In terms of severity, 122 out of the 127 students meeting the CVS definition (96%) had mild CVS (defined as a score of 6–12).

### 3.1. Determinants and predictors of developing computer vision syndrome

The univariate analysis showed that the mother’s educational level, employment status, and history of visual disturbances, were significantly associated with developing CVS. Higher proportions of participants with CVS were found among students of employed mothers compared to others (10.7% vs. 6.1%, *p* = 0.001) and among students with a history of visual disturbances compared to those without (19.6% vs. 4.7%, *p* < 0.001) The mean weekly screen time was significantly higher among students with CVS (33.1 ± 15.3 h/week), compared to those without (28.5 ± 14.0 h/week) with *p* < 0.001. No significant difference in the prevalence of CVS was found between males (8.8%), and females (7.6%), and between those 12–15 years of age (8.8%) and the younger participants (7.7%) (Table 2).

**Table 2:** Determinants and predictors of computer vision syndrome among included students.

Characteristics		Developed CVS No (%)	Value of p*	AOR (95%CI)	Value of *p*
Student’s age group	8–11 years	65 (7.7)	0.411	1.06 (0.72–1.57)	**0.769**
	12–15 years	62 (8.8)		1 [Reference]	
Sex	Female	59 (7.6)	0.371	0.71 (0.49–1.05)	0.085
	Male	68 (8.8)		1 [Reference]	
Nationality	Expatriate	83 (8.5)	0.566	1.02 (0.65–1.59)	0.942
	Local (Qatari)	44 (7.7)		1 [Reference]	
Number of siblings	3 or less	70 (9.5)	0.227	1 [Reference]	
	4–6	47 (7.2)		1.05 (0.68–1.60)	0.832
	>6	10 (6.6)		1.19 (0.52–2.70)	0.681
History of visual disturbances	No	55 (4.7)	**<0.001**	1 [Reference]	
	Yes	72 (19.6)		5.16 (3.48–7.65)	**<0.001**
Mother’s age category	<35	21 (7.1)	0.369	-------	-------
	35–44	83 (9.2)		-------	-------
	45–54	22 (7.3)		-------	-------
	55 or more	0 (0.0)		-------	-------
Father’s age category	<35	3 (4.4)	0.697	-------	-------
	35–44	58 (8.4)		-------	-------
	45–54	48 (8.5)		-------	-------
	55 or more	14 (7.8)		-------	-------
Mother’s education	No formal education	3 (3.9)	**0.018**	0.54 (0.13–2.24)	0.392
	Primary school level	7 (6.9)		1.03 (0.40–2.64)	0.949
	Preparatory school level	8 (6.3)		0.86 (0.36–2.10)	0.748
	Secondary/ high school level	27 (5.8)		0.68 (0.40–1.14)	0.143
	College or higher	82 (10.6)		1 [Reference]	
Father’s education	No formal education	3 (7.5)	0.95	1.49 (0.35–6.26)	0.589
	Primary school level	5 (6.5)		0.93 (0.33–2.61)	0.887
	Preparatory school level	8 (5.8)		0.73 (0.31–1.72)	0.475
	Secondary/ high school level	24 (5.8)		0.75 (0.44–1.29)	0.300
	College or higher	87 (9.9)		1 [Reference]	
Mother’s employment status	Employed	76 (10.7)	**0.001**	1.73 (1.14–2.65)	**0.011**
	Not employed	51 (6.1)		1 [Reference]	
Type of digital device that was used for online classes most of the time	Computers (desktops or laptops)	38 (9.6)	0.488	1 [Reference]	
	Notepad/iPad/Tablet	51 (7.7)		1.04 (0.63–1.72)	0.884
	Smartphone	38 (7.8)		0.80 (0.49–1.30)	0.361
Non-academic screen time (hours/week) (M ± SD)		33 ± 15	**<0.001**	1.02 (1.01–1.03)	**0.004**

M, mean; SD, standard deviation; AOR, adjusted odds ratio; CI, confidence interval. *Using Chi square or Fisher exact test as appropriate for categorical variables and independent *t*-test or Mann–Whitney test as appropriate for continuous and ordinal variables.

We conducted a multivariable logistic regression analysis to explore the potential predictors of developing CVS among students. The model was statistically significant when compared to the null model. In addition to clinically relevant variables, variables with *p* values of ≤2.5 on the univariate analysis were also included in the regression model. As shown in Table 2, we found that the mother’s employment, a positive history of visual disturbances, and the mean weekly screen time were significantly and independently associated with having CVS. Students of employed mothers were 1.7 times more likely to have CVS compared to others (AOR: 1.73, 95%CI: 1.14–2.65, *p* = 0.011). Those with a positive history of visual disturbances were about 5 times more likely to have CVS compared to those without (AOR: 5.16, 95%CI: 3.48–7.65, *p* < 0.001). For each additional hour in the average weekly screen time, the odds of having CVS increased by 2% (AOR: 1.02, 95%CI:1.01–1.03, *p* = 0.004).

## 4. Discussion

This study aimed to assess the prevalence of CVS during the periods of school closures and remote learning imposed by the COVID-19 pandemic, explore the associated factors, and present some digital health remedies that might reduce the risk. The study showed a prevalence of 8%, significant increase in the screen time, and found that mother’s employment, having positive history of visual disturbances, and excess screen time to be significant predictors of CVS.

The closure of educational institutions including schools to contain the spread of COVID-19 has forced countries to change the traditional face-to-face teaching methods into virtual online classes that require spending prolonged periods in front of the screens. Besides the online classes, children and adolescents started to engage more in using digital devices for leisure, and social communications like using social media (30). Excessive screen time has crucial negative impacts on the physical and mental health of children and adolescents. It can result in sleep and psychological disturbances, more sedentary behaviors with less physical activity, and can adversely affect ocular health (31).

The prevalence of CVS in this study was found to be approximately 8% which is much less than the prevalence reported in studies from Saudi Arabia, India, and Thailand, where about 35, 50, and 70% of children and adolescents met the definition of CVS using the same questionnaire, respectively (20, 23, 25). This might be explained by the higher mean age of the participants in those studies as they targeted high school children. This explanation can be supported by a recently published systematic review that assessed the correlates of screen time among children and adolescents and showed that older children had more screen time (32). Another explanation might be the timing of conducting those studies relative to ours, as those studies were conducted during schools’ closure, while we conducted the study after the reopening of schools and resuming the face-to-face learning by collecting the data retrospectively reflecting a higher chance of recall bias. Moreover, the study from Thailand relied on student-reported data while we collected the data from parents who might have not been fully aware of their children’s eye symptoms. Additionally, the same study relied on a different scoring method that overestimated the prevalence. Those studies used convenient sampling techniques and collected the data using online questionnaires which might have introduced selection bias. We expect that those who tend to complete online surveys are more likely to use digital devices than others who do not, and this might explain the higher prevalence in those studies. About 57% of students had one or more visual symptoms consistent to some extent with the results of other studies in Saudi Arabia and China (24, 33). The commonest symptom reported was headache like in previous studies (20, 23, 25, 34).

Expectedly, students of employed mothers were more likely to develop CVS consistent with the results of other studies (35, 36). This might be explained by the limited supervision of employed mothers on their children’s screen time while at work. However, it’s essential to consider the broader family and social dynamics. While our initial explanation leaned towards the notion that employed mothers might have limited oversight on their children’s screen time, it’s crucial to recognize the potential roles of other caregivers or family members. For instance, grandparents, older siblings, or even hired caregivers might step in to supervise or influence screen time.

During the pandemic, Qatar like other countries implemented working-from-home measures. Home became the new office for many mothers (1). By using telecommunication while working from home, parents might have unconsciously influenced the behaviors of their children and pushed them more toward using digital devices. Moreover, some working-from-home parents might have given their children digital devices to keep them occupied while they were having important online meetings (37). Students with a positive history of visual disturbances were about five times more likely to have CVS than those without. The American Optometric Association (AOA) stated that uncorrected or under-corrected vision problems can contribute to the development of CVS and computer-related eyestrain (21). Although most of the students with a positive history of visual disturbances in our sample were wearing eyeglasses or medical contact lenses, according to their parents, this does not mean that their vision problems were adequately corrected, and it does not give an idea of how compliant they were with wearing them. Moreover, eyeglasses or contact lenses prescribed for general use may not be adequate for computer work or digital screen use. Although in most cases, the CVS symptoms improve after stopping screen viewing, some individuals might continue to experience the persistence of some visual symptoms like blurred vision (21). Additionally, those facing difficulties in viewing digital screens due to some existing visual impairment might take uncomfortable odd postures, and improper ergonomics to see clearly which might precipitate or worsen musculoskeletal problems. Controlling the lighting and glare on the device screen, maintaining adequate distance and proper posture while viewing screens, and seeking medical care even for minor vision problems are critical strategies to prevent and minimize CVS as recommended by the AOA (21). The 20-20-20 rule, positing a 20 s break to observe an object 20 feet distant every 20 min during screen usage, is a prevalent guideline proposed to mitigate digital eye strain (31). Empirical evaluations of its efficacy yield varied outcomes. While certain investigations affirm its utility in attenuating DES and associated dry eye manifestations, others indicate its limited influence on specific visual metrics (38, 39). The prevailing consensus emphasizes the necessity for augmented research to validate the rule’s broad endorsement. Notably, the AOA endorses the 20-20-20 rule as a strategy to alleviate symptoms associated with CVS emphasizing its importance given the increasing duration individuals spend in front of digital screens (21).

Various digital health technologies can be utilized to reduce the risk of CVS. Eye exercise apps offer exercises that reduce eye strain, reminding students to take regular breaks (40). Blue light filters that decrease the amount of blue light emitted by screens and often promoted as mitigators of DES, have been extensively studied to determine their efficacy. While some studies indicate no significant alleviation of DES symptoms with the use of blue-blocking filters, others suggest a potential benefit (41, 42). However, the consensus is not unanimous, and the effectiveness of blue light filters remains a topic of ongoing investigation. It’s imperative for individuals and professionals to be informed of the diverse findings and make decisions based on a holistic understanding of the available evidence.

Screen brightness and contrast adjuster software programs automatically optimize screen settings for improved visual experience. Parents could utilize control apps and software that offer a range of features to help them establish healthy digital habits (43). Parents can set time limits on screen usage, schedule device-free periods, and even block access to certain apps or websites. Additionally, control devices provide options for monitoring and tracking screen time, allowing parents to gain insights into their children’s digital activities. Virtual eye exams allow students to conduct exams at home, identifying vision problems and providing recommendations. Through telemedicine, students can access virtual eye care services and receive professional guidance to mitigate the risk of CVS (44). Eye care professionals can remotely assess visual discomfort symptoms, provide personalized recommendations, and suggest proper visual hygiene practices. By addressing concerns and offering guidance through teleconsultation, students can effectively manage their screen time, adopt proper ergonomics, and practice regular eye exercises. This approach not only minimizes the adverse effects of prolonged digital device usage but also ensures that students receive timely eye care support despite physical school closures. Implementing these digital health technologies during school closures helps promote healthy screen habits and prevent long-term vision issues among students. These digital remedies serve as invaluable tools, empowering students to strike a harmonious balance between screen engagement and ocular well-being, ensuring a conducive learning environment throughout remote learning experiences.

This study provided insights into the prevalence of CVS among students during the period of shifting to online learning and associated factors in Qatar and shed the light on some strategies and digital health technologies that might help in reducing the risk of CVS during such times. We collected an adequate sample size and used an appropriate random sampling technique. Some of the drawbacks of this study are the timing of conducting it after the reopening of schools and relying on parents to report their children’s symptoms retrospectively which might have introduced some recall bias and underestimated the prevalence.

## 5. Conclusion

Schools’ closure and remote learning during COVID-19 significantly increased screen time and adversely impacted the vision of children and adolescents. The prevalence of CVS in our sample was found to be approximately 8%. Mother’s employment, positive history of visual disturbances, and excess screen time were significant predictors of CVS. Parents should pay more attention to their children’s digital device use. Healthcare providers in collaboration with teachers should provide parents with evidence-based strategies to prevent or minimize digital eye strain among students. Furthermore, it would be beneficial to conduct longitudinal studies to understand the persistence and evolution of CVS symptoms as students transition back to traditional learning environments. In the landscape of remote learning, the implementation of digital remedies emerges as a proactive approach to mitigate the risk of digital eye strain. Eye exercise apps, blue light filters, and screen optimization tools enhance visual comfort, while parental controls foster healthy digital habits. The rise of virtual eye exams and telemedicine ensures timely eye care, even in remote learning scenarios. These tools collectively safeguard students’ visual health, striking a balance between academic pursuits and ocular well-being.

## Data availability statement

The original contributions presented in the study are included in the article/supplementary material, further inquiries can be directed to the corresponding author.

## Ethics statement

The studies involving humans were approved by the institutional review board (IRB) of Hamad Medical Corporation two approvals were obtained one from the IRB of Hamad Medical Corporation and one from IRB of Primary Health Care Corporation. The studies were conducted in accordance with the local legislation and institutional requirements. Written informed consent for participation in this study was provided by the participants’ legal guardians/next of kin.

## Author contributions

MA: Conceptualization, Data curation, Formal analysis, Funding acquisition, Investigation, Methodology, Project administration, Resources, Supervision, Validation, Writing – original draft, Writing – review & editing. SA: Data curation, Formal analysis, Investigation, Methodology, Validation, Writing – original draft. NS: Supervision, Writing – review & editing. LA: Supervision, Writing – review & editing. IB: Supervision, Writing – review & editing.
